# Supplementary material for: Identifying (un)controllable dynamical behavior in complex networks
Source: PLoS Comput Biol. 2018 Dec 10;14(12):e1006630. doi: 10.1371/journal.pcbi.1006630 (PMC6301693; doi:10.1371/journal.pcbi.1006630)
Supplement: S1 Appendix — (PDF) [file pcbi.1006630.s001.pdf]

Supplemental Material

October 31, 2018

1 Boolean Stable Motifs

Here, we expand upon the concepts discussed in [22, 30]. A Boolean network is a discrete-time system composed of variables or nodes $X_i(t)$ that can take values in $\{0,1\}$ along with update functions $f_i = f_i(\{X_j\})$. At each time-step, a subset of $\{X_i(t)\}$ is updated according to $X_i^* = f_i(\{X_j\})$, where X_i^* denotes the updated value of $X_i(t)$. The two most commonly considered update schemes are synchronous update, in which all nodes are updated at each time-step, or general asynchronous update, in which only one randomly selected node is updated at each time-step.

The expanded network of a Boolean system is built by first constructing a pair of virtual nodes for each Boolean variable corresponding to its two states. In a slight abuse of notation, we denote these nodes X_i and $\neg X_i$. To proceed, we note that the Boolean update functions can be written in disjunctive normal form (DNF)

$$f_i = \bigvee_{j=1}^{N}\left(\bigwedge_{m=1}^{M_j} X_{l_m} \bigwedge_{q=1}^{Qj} \neg X_{p_q}\right). \tag{1.1}$$

The negation $\neg f_i$ of each Boolean function can also be written in DNF. Each term of the form $\bigwedge_{m=1}^{M_j} X_{l_m} \bigwedge_{q=1}^{Qj} \neg X_{p_q}$is called a prime implicant of f_i and either corresponds to an existing virtual node, or else we identify the term with a composite node and draw edges from each factor X_{l_m} and $\neg X_{p_q}$ to the composite node. From each node in the expanded network that corresponds to a prime implicant of f_i (or $\neg f_i$), we draw an edge to the virtual node X_i (or $\neg X_i$). This scheme implies the following: if there is an edge in the expanded network from $\bigwedge_{m=1}^{M_j} X_{l_m} \bigwedge_{q=1}^{Qj} \neg X_{p_q}$ to X_i, then whenever $\bigwedge_{m=1}^{M_j} X_{l_m} \bigwedge_{q=1}^{Qj} \neg X_{p_q} = 1$ holds, X_i will always update to 1 in the next update step. Similarly, if there is an edge from $\bigwedge_{m=1}^{M_j} X_{l_m} \bigwedge_{q=1}^{Qj} \neg X_{p_q}$ to $\neg X_i$ and $\bigwedge_{m=1}^{M_j} X_{l_m} \bigwedge_{q=1}^{Qj} \neg X_{p_q} = 1$ holds then X_i will update to 0 (i.e., $\neg X_i$ becomes 1). Thus in either case, the edge is both a maintenance and driving edge. As such, the Boolean expanded network, as described above, is an expanded network of the type described in the main text. It is also noteworthy that the update functions f_i can be reconstructed from the expanded network.

A stable motif in the expanded network is a subgraph S with the following properties: (i) all nodes in S have a parent (regulator) in S or else have a self-loop, (ii) S does not include any contradictory nodes (e.g., both X_i and $\neg X_i$), (iii) S includes all the parents (regulators) of included composite nodes, and (iv) S has no proper subgraphs satisfying the first three properties (note that condition (iv) implies that S is strongly connected). We call subgraphs satisfying the first three conditions *stable modules.* Such network components correspond to values of the Boolean variables that are stable, independent of the rest of the system.

As an example, consider the following Boolean network:

$$\begin{aligned} f_A &= A \wedge B \\ f_B &= A \vee C \\ f_C &= A \wedge B \vee \neg C \end{aligned} \tag{1.2}$$

For convenience, we give the negated update functions as well:

$$\begin{aligned} \neg f_A &= \neg A \vee \neg B \\ \neg f_B &= \neg A \wedge \neg C \\ \neg f_C &= (\neg A \wedge C) \vee (\neg B \wedge C). \end{aligned} \tag{1.3}$$

We can now construct the expanded network, as the update rules and their negations are written in DNF in Equations 1.2 and 1.3. By inspecting these rules, as written, we see that in addition to the virtual nodes A, B, C, $\neg A$, $\neg B$, and $\neg C$, we will need to construct composite nodes $A \wedge B$, $\neg A \wedge \neg C$, $\neg A \wedge C$, and $\neg B \wedge C$, for a total of ten nodes. We draw edges to the composite nodes from each of their factors (e.g., there are edges from A and B to $A \wedge B$). We also draw edges to the virtual nodes according to the prime implicants of the update rules (e.g., there are edges from A and C to B, and edges from $\neg A \wedge C$, and $\neg B \wedge C$ to $\neg C$). The completion of this procedure yields the full Boolean expanded network.

We graphically represent the network given by Equations 1.2, construct its expanded network, and identify stable modules and stable motifs in Figure 1.1. This network has two attractors. The first is the steady state in which all variables are true. The second is the one in which A is false, and B and C oscillate. If A is ever false, it stays false. Plugging $A = 0$ into the update rules for B and C gives $f_B = C$, $f_C = \neg C$, so C oscillates. The timing of when C is true and when it is false depends on the update scheme, but the update scheme does not change the fact that C oscillates. Node B follows C; thus it oscillates as well. If A is true at some late time, it is not oscillating (because $A = 0$ self-stabilizes). If $A = 1$ is stabilized, it can only be because $B = 1$ is also stabilized, which requires

$C = 1$ as well. This can also be seen from the fact that the only solution of the steady state equations $A = A \wedge B$, $B = A \vee C$, and $C = A \wedge B \vee \neg C$ is $A = B = C = 1$. Because these attractors are characterized only by the value of A, there can only be two attractors (one for each value). We further note that the stable motifs that characterize these attractors are independent of update scheme (this is a general feature of stable motifs in Boolean systems [30]). There are two stable motifs, each of which is a stable module, and node C can be added to one of the motifs to create a larger stable module. The first consists of only $\neg A$, and this stable motif captures the behavior that if A ever becomes false, it remains false. The other two stable modules both contain A, B, and $A \wedge B$. One also contains C. The A, B, and $A \wedge B$ motif indicates that if A and B are both true, they both remain true, independent of C (e.g., if $C = 0$ were held fixed by some external control, $A = B = 1$ would remain stable). The stable module that also contains C identifies $A = B = C = 1$ as a steady state of the system.

2 Dynamical Properties of Biomolecular Systems

The dynamics of biomolecular systems are almost always bounded (i.e. the within-cell concentrations of proteins or molecules do not increase without bound). Furthermore, most biomolecular interactions and direct regulatory relationships are monotonic; it is infrequent that a regulator such as a transcription factor or enzyme directly inhibits certain processes (transcription or reactions) and activates others. In many cases, apparent non-monotonic relationships arise from the effects of as-of-yet unknown mediators. This situation is likely in biomolecular networks, in which regulatory relationships are often not mapped to the level of elementary reactions. For example, in considering the regulatory relationship $\dot{x} = w^3 - w - x$, we might at first believe that w is a non-monotonic regulator of x, when in reality, a more accurate model of the system might be $\dot{x} = y - w - x$, $\dot{y} = \alpha \left(w^3 - y\right)$, which consists of an incoherent feed-forward loop involving monotonic regulation. This example is especially believable when $\alpha \gg 1$, because y will respond extremely quickly to changes in w. There is typically only one additional variable required per instance of non-monotonic regulation. We note that even when a hidden variable cannot be biologically motivated, it may still be introduced without loss of fidelity in order to make the relationships in the model monotonic.

For these reasons, we focus on ODE systems that take the form

$$\dot{x}_i = F_i\left(\boldsymbol{x}\right), \tag{2.1}$$

where F_i is continuous, monotonic in each of its arguments, and bounded.

3 Mathematical foundations of expanded networks

3.1 Expanded Networks and Stable Modules

In this section, we present the key definitions and results of the main text in the more formal style of dynamical systems theory. Throughout this section, we consider a dynamical system (T, M, Φ), where Φ is a monoid action of T on a state space M. That is, T is a set representing the evolution variable (e.g., time), and we place an evolution operator $+$ on T that is associative and for which T has an identity element, e. The space M plays the role of the phase space or state space. The monoid action Φ describes the evolution of the system by mapping a point $x \in M$ and a "time" $t \in T$ to a new point in M, which is denoted by $\Phi^t(x)$. This function must obey $\Phi^a\left(\Phi^b(x)\right) = \Phi^{a+b}(x)$ so that Φ is consistent with the notion of time evolution. It can be given explicitly (as in Boolean networks, where Φ is the time-explicit update function) or implicitly (as in ODE systems, in which Φ is given by the solutions to the ODEs for each initial condition). In the main text, we focus on the case in which T is one-dimensional and M is finite-dimensional, however, the contents of this section apply generally.

To apply our methods, we must have a rule for determining whether or not one "time" follows another. To do this we define a total preorder on a submonoid S of T denoted by $\lesssim$ and satisfying $e \lesssim s$ for all $s \in S$, where $e \in S$ is the identity. A total preorder is, by definition, a transitive relation for which either or both $x \lesssim y$ or $y \lesssim x$ hold.

Of particular interest here is the case of autonomous first order ODE systems, in which T is $\mathbb{R}^+(+)$, M is a box in $\mathbb{R}^N$, Φ is the flow of the defining differential equations, and we take $S = T$. We also consider Boolean systems, in which T is $\mathbb{Z}^+(+)$, M is $\{0,1\}^N$, Φ is the Boolean update function, and we again take $S = T$.

The objects of primary importance in the main text are expanded networks, which are constructed for sets of statements regarding the values of variables. These statements may be viewed as subsets of M. To define these networks requires that we have a more general notion of what it means for a trajectory to be confined to a region for a particular duration. We thus define the following sets:

Definition 1. The *persistence set* of a point r in a subset R of M is $P(R, r) = \{s_0 \in S : s \lesssim s_0 \implies \Phi^s(r) \in R\}$.

Each element of the persistence set is a time for which the trajectory starting at r is confined to R. Note that e is always an element of $P(R, r)$, so the persistence set is never empty. With the above shorthand, we can formally define the maintenance relation as follows:

Definition 2. For non-disjoint subsets R_1 and R_2 of M, we say R_1 *maintains* R_2 under S (denoted $R_1 \to R_2$) if for each $r \in R_1 \cap R_2$, $P(R_1, r) \subseteq P(R_2, r)$ holds with equality holding only when $P(R_1, r)$ and $P(R_2, r)$ are equal to S.

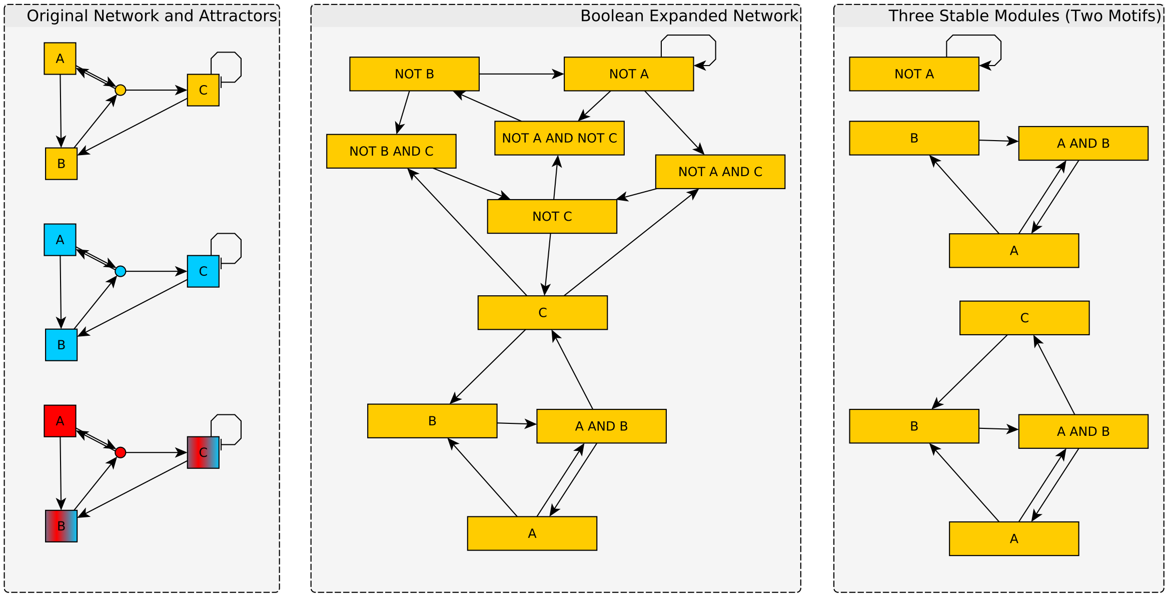

Figure 1.1: From left to right: (i) schematic of the network from Equations 1.2 with its two attractors (blue is true, red is false, and the gradient represents oscillation) (ii) the full expanded network of Equations 1.2 (iii) the three stable modules that exist in the network. The "NOT A" stable module (motif) captures the behavior that if A ever becomes false, it remains false. The other two stable modules identify the steady-state attractor of the system.

Informally, the above definition means that R_1 maintains R_2 if any trajectory starting in $R_1 \cap R_2$ that is confined to R_1 for a certain duration is confined to R_2 for a strictly longer duration. The maintenance relation induces a directed graph on any collection of regions of M. We next develop the notion of expanded networks in the special case in which there are no composite nodes. We then prove the stabilization results of the main text for this special case. We later show that the case in which composite nodes are present follows as a simple extension of that result.

Definition 3. The *composite-free expanded network* for a collection $\mathscr{R}$ of regions in M is the directed graph $\mathcal{G}$ with vertex set $\mathscr{R}$ and edges given by the maintenance relation.

We denote the intersection of all regions in a subgraph G of $\mathcal{G}$ by $I(G)$.

Definition 4. A subgraph G of $\mathcal{G}$ is *source-free* if for each node R_j in G, there is an edge $R_i \to R_j$ in G for some R_i in G. A subgraph G of $\mathcal{G}$ is *consistent* if the intersection of all regions represented in G, $I(G)$, is non-empty.

This leads to the definition of a stable module for a composite-free expanded network and to a key result about composite-free expanded networks.

Definition 5. A finite, source-free subgraph G of a composite-free expanded network $\mathcal{G}$ with $I(G)$ nonempty is called a *(composite-free) stable module* of $\mathcal{G}$ with *trap space* $I(G)$.

Theorem 6. *The trap space, $I(G)$, of a (composite-free) stable module G of $\mathcal{G}$ is closed under S, i.e., for any $s \in S$, $\Phi^s(I(G)) \subseteq I(G)$ holds.*

Proof. Because G is source-free, each node in G is reachable from some cycle in G. Suppose $C_1 \to C_2 \to \ldots \to C_N$ forms a cycle in G. Consider any $r \in I(G)$. Because no $P(C_i, r)$ can be its own proper subset it must be that $P(C_i, r) = S$ holds for all regions C_i that are part of any cycle. Because $R_1 \to R_2$ implies $P(R_1, r) \subseteq P(R_2, r)$, it follows that $P(R_i, r) = S$ holds for all $R_i \in V(G)$. ☐

In order to fully align with previous work in Boolean systems, we require a formal notion of the "composite nodes" discussed in the main text. To motivate the definition of these composite nodes here, consider the following remark:

Remark. If $R_{a_i} \to R_{b_i}$ holds for finite sequences a_i and b_i and $\bigcap_i (R_{a_i} \cap R_{b_i})$ is non-empty, then $\bigcap_i R_{a_i} \to \bigcap_i R_{b_i}$ holds also.

Proof. Take $r \in \bigcap_i (R_{a_i} \cap R_{b_i})$. The case in which $P\left(\bigcap_i R_{a_i}, r\right) = \phi$ is vacuous. Hence, take $P\left(\bigcap_i R_{a_i}, r\right)$ non-empty. Note that $P\left(\bigcap_i R_{a_i}, r\right)$ is equal to $\bigcap_i P(R_{a_i}, r)$. This implies that $P\left(\bigcap_i R_{a_i}, r\right)$ is a subset of $P\left(\bigcap_i R_{b_i}, r\right)$. Because the sequence of maintenance relations is finite, we may choose R_{a_k} such that $P(R_{a_k}, r) \subseteq P(R_{a_i}, r)$ holds for all R_{a_i}, and thus $P\left(\bigcap_i R_{a_i}, r\right) = P(R_{a_k}, r)$ holds. We can similarly choose R_{b_m} such that $P\left(\bigcap_i R_{b_i}, r\right) = P(R_{b_m}, r)$ holds. If $P(R_{a_k}, r) =$

$P(R_{b_m}, r)$, then $P(R_{a_m}, r) = P(R_{a_k}, r) = S$ follows from the fact that $P(R_{a_k}, r) \subseteq P(R_{a_m}, r)$ and $R_{a_m} \to R_{b_m}$ both hold. □

With this in mind, we introduce the notion of composite nodes as a shorthand for considering intersections of regions. Suppose that $\bigcap_i R_{b_i} \to R$ holds for some R, but that each R_{b_i} is already under separate consideration. By the previous remark, $\bigcap_i R_{b_i}$, and thus R, will be maintained if each R_{b_i} is maintained. If $\bigcap_i R_{b_i}$ is considered explicitly as a region, then the intersections of regions that maintain each R_{b_i} must also be considered explicitly and separately (and the regions that maintain these must then be considered, etc.). To avoid this, we introduce the composite node $C(\{R_{b_i}\})$:

Definition 7. For a finite sequence of regions $\{R_{b_i}\}$, the *composite node* or *composite region* is denoted $C(\{R_{b_i}\})$. The regions R_{b_i} are called the *factors* of $C(\{R_{b_i}\})$. We say that $C(\{R_{b_i}\})$ maintains a region R of M if and only if $\bigcap_i R_{b_i} \to R$ holds.

With this definition, we are now able to define the expanded network as discussed in the main text:

Definition 8. For a given collection $\mathscr{R}$ of regions in M and collection $\mathscr{C}$ of composite nodes with factors in $\mathscr{R}$, the *expanded network* $\mathscr{G}$ is the directed graph on the node-set $V(\mathscr{G}) = \mathscr{C} \cup \mathscr{R}$ with edges placed according to the following two rules: i) if $R \in \mathscr{R}$ is maintained by $V \in V(\mathscr{G})$, then (V, R) is an edge of $\mathscr{G}$ ii) if $C \in \mathscr{C}$ has a factor $R \in \mathscr{R}$, then (R, C) is an edge in $\mathscr{G}$.

Definition 9. Let G be a subnetwork of an expanded network $\mathscr{G}$. G is *source-free* if every node of G has a parent node in G. G is *consistent* if the intersection of all regions, including those represented by composite nodes, in G is non-empty (i.e., $I(G)$ is non-empty). G is *complete* if the factors of each composite node in G are also in G; if G is complete then G is also an expanded network. A source-free, consistent, and complete expanded (sub) network is called a stable module.

Corollary 10. *If an expanded network $\mathscr{G}$ is a stable module, then $I(\mathscr{G})$ is closed under S.*

Proof. Let $\mathscr{G}$ have composite nodes $\mathscr{C}$ and regions $\mathscr{R}$. Let $\mathcal{R}$ be the set of all possible intersections of regions in $\mathscr{R}$ and let $\mathcal{G}$ be the composite-free expanded network constructed on $\mathcal{R}$. Note that because $\mathscr{G}$ is complete and consistent, $I(\mathcal{G}) = I(\mathscr{G})$ is non-empty. Consider any $\bigcap_i R_{b_i} \in \mathcal{R}$. Because $\mathscr{G}$ is source-free, each R_{b_i} has at least one parent region R_{a_i} for which $R_{a_i} \to R_{b_i}$ holds, and $\bigcap_i R_{a_i} \in \mathcal{R}$ maintains $\bigcap_i R_{b_i}$. Therefore, the composite-free expanded network $\mathcal{G}$ constructed on $\mathcal{R}$ is source-free and consistent. Thus $\mathcal{G}$ is a composite-free stable module, implying that $I(\mathcal{G}) = I(\mathscr{G})$ is closed under S. □

It is important to note that the expanded network for a dynamical system is not unique. Rather, a collection of regions and composite nodes must first be specified. After this is done, the expanded network is uniquely defined, but in practice, this network need not be fully identified. It suffices to find the edges between regions and composite nodes that form a stable module, which yields a trap space. The identification of additional edges cannot invalidate the closure of the resulting trap space under S, although these added edges may lead to the discovery of smaller trap spaces contained within the initial trap space. Therefore, trap spaces can be discovered by identifying stable modules without explicitly constructing an expanded network that the stable module is embedded within.

3.2 Worst case systems

To motivate the use of "worst-case" subsystems, as used in the algorithm presented in the main text for biological ODE systems, consider a family of dynamical systems $\{(T, M, \Phi_f) : f \in F\}$ with a shared submonoid S. We allow f to be a discrete or continuous index (i.e., F can be any set). If the maintenance relations of a stable module G hold for all members of the family, then straightforward application of the stable module theorem indicates that the corresponding trap space of the stable module is closed under each Φ^S_f. If a total preorder $\lesssim$ can be placed on F such that $f_1 \lesssim f_2$ and $\Phi^S_{f_1}(I(G)) \subseteq I(G)$ imply that $I(G)$ is closed under $\Phi^S_{f_2}$, then it suffices to show that $\Phi^S_{f_{min}}(I(G)) \subseteq I(G)$ holds for any f_{min} satisfying $f_{min} \lesssim f$ for all $f \in F$. This is the concept underlying the selection of a "worst-case" system. The actual system under consideration together with all externally controlled versions of that system are taken to form $\{(T, M, \Phi_f) : f \in F\}$, and the worst-case system is one described by the monoid action $\Phi^S_{f_{min}}$. In our applications, F is the set of fixed regulatory effects and their values, and $f_1 \lesssim f_2$ only if for all initial conditions r in a candidate stable module, $\Phi^S_{f_1}(r)$ has a smaller minimum distance to each of the candidate thresholds than $\Phi^S_{f_2}(r)$ does (informally, Φ_{f_1} is "closer to violating" the candidate stable module). Rather than explicitly constructing $\{(T, M, \Phi_f) : f \in F\}$, we rely on the properties of monotone systems to identify the ODEs governing $\Phi_{f_{min}}$ directly, as is described in the main text.

4 Sign-Consistent Networks

Following Sontag [20], sign-consistent networks are those to which we can assign $\sigma_i = \pm 1$ to each node of the associated regulatory network such that the product $\sigma_i \sigma_j$ is 1 whenever nodes i and j are connected by a positive edge, and -1 whenever they are connected by a negative edge. Such networks represent systems for which any effect of one variable on another is unambiguously monotonic, implying a lack of negative feedback loops and incoherent feed-forward loops. These notions are equivalent: in a graph that lacks negative cycles and feed-forward loops,

starting from any node of each weakly connected component, one may assign $\sigma_i = 1$ and perform a breadth-first traversal ignoring edge direction, assigning subsequent σ_i values by alternating sign whenever a negative edge is used.

Consider the subnetwork of the regulatory network which corresponds to a candidate stable module, S_c (which, as described in the main text, is source free, composite-closed, and consistent). The nodes of this subnetwork are the nodes characterized by the statements that make up the virtual nodes of S_c, and the edges of the subnetwork determine the maintenance or driving edges among virtual nodes of S_c. Importantly, edges among the nodes of this subnetwork that do not contribute to the stable module are not included in this subnetwork. It is not difficult to realize that a subnetwork defined this way is sign-consistent. Indeed, variables represented by virtual nodes of the form $x_i < T_i^\alpha$ can be assigned $\sigma_i = -1$ and all others can be assigned $\sigma_i = 1$. This is possible because the only sources of negative regulation in the subnetwork arise from nodes of this first type, which, due to the consistency of S_c, always contribute pairs of negative edges to S_c and the associated modified regulatory subnetwork.

5 Example: Identification of a Stable Module

Consider the system

$$\begin{aligned}
\dot{w} &= H(z) - w \\
\dot{x} &= H(w) - x \\
\dot{y} &= H(x) - y \\
\dot{u} &= x - u \\
\dot{z} &= 1 + H(y)H(u) - H(u) - z \\
H(\cdot) &= \frac{[\cdot]^2}{[\cdot]^2 + 1/4}.
\end{aligned} \tag{5.1}$$

By inspection, we note that the following structure is present in the expanded network, for unspecified thresholds:

$$(z > T_z) \to (w > T_w) \to (x > T_x) \to (y > T_y). \tag{5.2}$$

We therefore conjecture the existence of a stable module that includes this structure and $(y > T_y) \to (z > T_z)$. The only edge in the regulatory network incident to a variable represented in this candidate stable module that is not represented in this candidate stable module is the regulation of z by u. This is a negative edge because $H(y) < 1$, and makes the network sign-inconsistent. To search for valid thresholds for this candidate stable module, we fix the effect of this edge at the "worst case" value. In this example, the worst case corresponds to the value of u that most greatly inhibits z. Because u will be bounded by $\sup x = \sup H(w) = 1$, that value is $u = 1$. This yields the following modified system:

$$\begin{aligned}
\dot{w} &= H(z) - w \\
\dot{x} &= H(w) - x \\
\dot{y} &= H(x) - y \\
\dot{z} &= 1 + H(y)H(1) - H(1) - z.
\end{aligned} \tag{5.3}$$

Note that this is a monotone, sign-consistent system because we are holding the sign-inconsistent regulation fixed. We now find the steady states of this modified system following the MIOS procedure outlined in [20, 29]. The steady states correspond to the roots of the function feedback function

$$k(z) = 1 + H(H(H(H(z))))H(1) - H(1) - z, \tag{5.4}$$

which is obtained by considering the regulation of (e.g.,) z by its inputs when the regulatory effect of z (on w) is held fixed (i.e., w will approach $H(z)$, so x will approach $H(H(z))$, and so on until z approaches $k(z) + z$). There are three roots of this equation in the interval $(0, 1)$. We consider the largest root, which we call r and find numerically to be $r = 0.68$, though the following discussion is equally valid with either other root used in place of r. The modified subsystem has a steady state

$$w = H(r),\ x = H(H(r)),\ y = H(H(H(r))),\ z = r, \tag{5.5}$$

and so $(z > r) \to (w > H(r)) \to (x > H(H(r))) \to (y > H(H(H(r)))) \to (z > r)$ is a stable module (we may, by inspection of the original system, extend the module to include $(x > H(r)) \to (u > H(r))$).

We interpret this result as follows. If $w, x, y > H(r)$ and $z > r$ hold at any time, then these will continue to hold for all time. This is true even if u is externally controlled, provided u remains smaller than one. If we are unsatisfied with this restriction on u, we may instead take the "worst-case" value for u to be infinity (rather than one), in which case the resulting characteristic has only one root, 1/2, in $(0, 1)$; this corresponds to the stable motif $\left(z > \frac{1}{2}\right) \to \left(w > \frac{1}{2}\right) \to \left(x > \frac{1}{2}\right) \to \left(y > \frac{1}{2}\right) \to \left(z > \frac{1}{2}\right)$, which has a similar interpretation.

6 Example: Comparison of Phase-Portrait and Stable Module Analyses

We consider the system given in Equations 6.1:

$$\begin{aligned}
\dot{x} &= 1 - H(y) - x, \\
\dot{y} &= H(z)(1 - H(x)) - y
\end{aligned} \tag{6.1}$$

where $H(x) = \frac{x^2}{x^2 + 1/16}$. In this example, $z = z(t)$ is not specified beyond being constrained to the interval $[0, 1]$. In this section, we will analyze this system using a traditional visual approach (phase portraits) and compare with the results obtained using the methods of the main text. To study this system using phase portraits requires a straightforward adjustment to account for the presence of

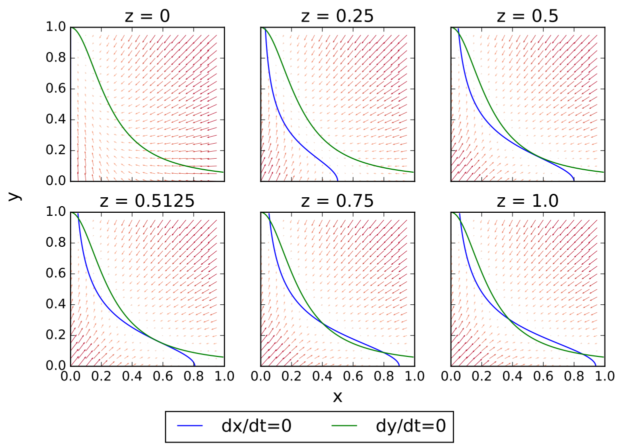

Figure 6.1: Phase-portrait slices and nullclines of the system given in Equations 6.1. Because $z = z(t)$ is not fixed, the system trajectories can travel between slices. We have chosen to display $z = 0.5125$ in addition to the regularly spaced examples, as the number of intersections of the nullclines changes at approximately this value (there is only one intersection each in the $z = 0$, $z = 0.25$, and $z = 0.5$ panels, and there are three intersections each in the $z = 0.75$ and $z = 1.0$ panels).

the unknown function z: we examine only the projection of each vector of the vector field onto the xy-plane, with the understanding that the z-component of system trajectories varies in some unknown way. To visualize the resulting three-dimensional phase portrait, we produce slices of constant z. In Figure 6.1 we show cross-sections at equal intervals, and also at $z = 0.5125$, as this is approximately the value at which the number of intersections of x and y nullclines in each slice changes (these intersections do not necessarily correspond to equilibria, as z is not constant).

We can interpret this series of phase-portrait cross-sections as a collection of phase portraits for systems in which z is held fixed as a constant parameter. Despite the lack of information regarding z, we can extract several conclusions from these figures. For example, we see that there is always an intersection of nullclines in which x is low (below about 0.1) and y is high (above about 0.95). One may hypothesize that x being low and y being high is robust to variations in z and test this hypothesis through careful examination of the vector field in a box bounding all of these qualitatively similar nullcline intersection points. Alternatively, one may follow our proposed methods.

The first step is to identify the causal relationships between variables. For example, if y is less than or greater than some threshold for sufficiently long, this will drive x above or below $1 - H(y)$. In the language of virtual nodes, this statement implies that any virtual node $y \lessgtr T$ has an edge to $x \lessgtr 1 - H(T)$ in the expanded network. It is therefore reasonable to study candidate stable modules of the form $y \lessgtr T_1 \leftrightarrow x \gtrless T_2$. Note that the direction of the inequalities is opposite for the two statements; this is a general feature of stable modules supported by mutual inhibition feedback loops.

We consider that $z(t)$ varies between 0.5125 and 1, corresponding to the region of bistability for fixed $z(t)$ (Figure 6.1). We can analyze this case by considering the "worst-case" system for each of our two candidate stable modules (one candidate has $y > T_1$ and $x < T_2$, while the other has the inequalities reversed). The construction of the worst-case system relies on the observation that an increase (decrease) in z increases (decreases) y, while decreasing (increasing) x.

In one stable module candidate, x is bounded below while y is bounded above. A decrease in z perturbs x and y toward violation of these bounds. Therefore, the worst-case system for the first candidate stable module is characterized by $\dot{y} = H(0.5125)(1 - H(x)) - y$ and $\dot{x} = 1 - H(y) - x$. The steady state of this worst-case system with the lowest value of x (and thus the highest value of y) is given by $x \approx 0.16$ and $y \approx 0.58$. Therefore, one stable module for this system is $x < 0.16 \leftrightarrow y > 0.58$. Note that this corresponds to an intersection of nullclines in the $z = 0.5125$ panel of Figure 6.1. In the other candidate stable module, x is bounded below and y is bounded above. In this case, the worst-case system is characterized by a large value of z, i.e., we set z to its maximum value, one. We then find the stable module $x > 0.96 \leftrightarrow y < 0.052$ from the steady state of this worst-case system with the largest value of x (and thus lowest value of y). These two stable modules describe positive invariant sets (i.e., trap spaces) that are valid for as long as z remains in the interval $[0.5125, 1]$. We may uncover less restrictive (i.e., bigger) trap spaces by considering the other steady states of the two worst-case systems we have already constructed; any steady state of a worst case system provides valid bounds for a candidate stable module.

In this section, we have used traditional techniques alongside the methods of the main text to analyze the system of Equations 6.1. We wish to emphasize that traditional visual methods, such as phase portraits and bifurcation diagrams become cumbersome as the system dimension becomes large, whereas the difficulty in solving the inequalities required for identifying thresholds for each candidate stable module scales linearly with the subsystem dimension. The crux of the method of our approach is to identify a simple "worst-case" subsystem that can be easily analyzed, and to pick out properties of the worst-case subsystem that are preserved within the real system. In this way, we can study a large, complex system by bounding the behavior of its simpler constituent subsystems.

7 Parameters and Variables for the Segment Polarity Model

Using the model and notation of [35], equations (3) and (11)-(23) the parameters we have used in our considera-

tion of the *Drosophila* segment polarity gene regulatory network are as follows:

For all time constants (H): $H = 1$

For all Hill functions (Φ): $K = 0.25$, $\nu = 2$

For all modified regulatory functions (Ψ): $K = 0.5$, $\nu = 4$, $a_0 = 0.1$

For *wingless* diffusion: $C_0 = 0.5$, $K_{in,WG} = K_{out,WG} = 0.05$

In addition, we provide the full names of the variables for convenience in Table 7.1.

Variable	Description
en	*engrailed* mRNA
EN	*engrailed* protein
sp	*sloppy-paired* mRNA
SP	*sloppy-paired* protein
hh	*hedgehog* mRNA
HH	*hedgehog* protein, internal
H_{nbr}	*hedgehog* protein, neighboring cells
ci	*cubitus interuptus* mRNA
CA	*cubitus interuptus* protein, activating form
CR	*cubitus interuptus* protein, repressing form
wi	*wingless* mRNA
WI	*wingless* protein, internal
WE	*wingless* protein, external
E_{nbr}	*wingless* protein, neighboring cells

Table 7.1: Table of variable abbreviations for the *Drosophila* segment polarity gene regulatory network of [35].
