# Supplementary material for: Identifying (un)controllable dynamical behavior in complex networks
Source: PLoS Comput Biol. 2018 Dec 10;14(12):e1006630. doi: 10.1371/journal.pcbi.1006630 (PMC6301693; doi:10.1371/journal.pcbi.1006630)
Supplement: S1 Fig — (PDF) [file pcbi.1006630.s003.pdf]

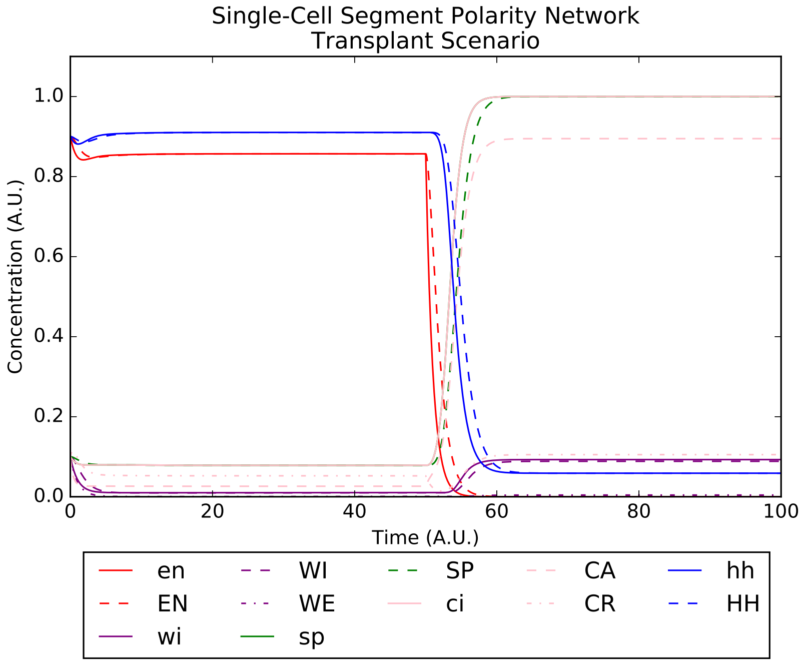

Supplemental Figure 1: Numerical simulation of the *Drosophila* segment polarity network cell-transplant experiment described in the main text. Abbreviations and parameter values are those described in section 6 of S1 Appendix. At $t = 0$, the neighboring cells express high *wingless* and low *hedgehog* ($E_{nbr} = 1$ and $H_{nbr} = 0$). The subject cell begins in an initial state consistent with cell type PC1 (high *engrailed* and *hedgehog*), and quickly approaches the associated steady state. At $t = 50$, the expression levels of E_{nbr} and H_{nbr} are switched, and the PC1 steady state can no longer be maintained. The system evolves toward a steady state associated with the PC2 cell type (high *sloppy-paired* and *cubitus interuptus*), in agreement with the arguments made in the main text.
